# A randomized controlled pilot trial comparing the impact of access to clinical endocrinology video demonstrations with access to usual revision resources on medical student performance of clinical endocrinology skills

**DOI:** 10.1186/1472-6920-13-135

**Published:** 2013-10-03

**Authors:** Emily J Hibbert, Tim Lambert, John N Carter, Diana L Learoyd, Stephen Twigg, Stephen Clarke

**Affiliations:** 1 Sydney Medical School Nepean, University of Sydney, PO Box 63, Penrith, NSW 2751, Australia; 2 Nepean Hospital, Penrith, Australia; 3 Sydney Medical School Concord, University of Sydney, Clinical Sciences Building, Concord Hospital, Concord, NSW 2139, Australia; 4 Brain and Mind Research Institute, University of Sydney, 100 Mallett Street, Camperdown, NSW 2050, Australia; 5 Hornsby Hospital, Palmerston Rd, Hornsby, NSW 2077, Australia; 6 Sydney Medical School, The University of Sydney, Sydney, NSW 2006, Australia; 7 Sydney Medical School Northern, University of Sydney, Sydney, Australia; 8 Department of Endocrinology, Royal North Shore Hospital, Pacific Highway, St Leonards, NSW 2065, Australia; 9 Sydney Medical School Central, University of Sydney, Sydney, Australia; 10 Department of Endocrinology, Royal Prince Alfred Hospital, Missenden Rd, Camperdown, NSW, 2050, Australia

**Keywords:** Video, Clinical skills, Medical student, Endocrinology, Medical education, E-learning

## Abstract

**Background:**

Demonstrating competence in clinical skills is key to course completion for medical students. Methods of providing clinical instruction that foster immediate learning and potentially serve as longer-term repositories for on-demand revision, such as online videos demonstrating competent performance of clinical skills, are increasingly being used. However, their impact on learning has been little studied. The aim of this study was to determine the value of adjunctive on-demand video-based training for clinical skills acquisition by medical students in endocrinology.

**Methods:**

Following an endocrinology clinical tutorial program, 2^nd^ year medical students in the pre-assessment revision period were recruited and randomized to either a set of bespoke on-line clinical skills training videos (TV), or to revision as usual (RAU). The skills demonstrated on video were history taking in diabetes mellitus (DMH), examination for diabetes lower limb complications (LLE), and examination for signs of thyroid disease (TE). Students were assessed on these clinical skills in an observed structured clinical examination two weeks after randomization. Assessors were blinded to student randomization status.

**Results:**

For both diabetes related clinical skills assessment tasks, students in the TV group performed significantly better than those in the RAU group. There were no between group differences in thyroid examination performance. For the LLE, 91.7% (n = 11/12) of students randomized to the video were rated globally as competent at the skill compared with 40% (n = 4/10) of students not randomized to the video (p = 0.024). For the DMH, 83.3% (n = 10/12) of students randomized to the video were rated globally as competent at the skill compared with 20% (n = 2/10) of students not randomized to the video (p = 0.007).

**Conclusion:**

Exposure to high quality videos demonstrating clinical skills can significantly improve medical student skill performance in an observed structured clinical examination of these skills, when used as an adjunct to clinical skills face-to-face tutorials and deliberate practice of skills in a blended learning format. Video demonstrations can provide an enduring, on-demand, portable resource for revision, which can even be used at the bedside by learners. Such resources are cost-effectively scalable for large numbers of learners.

## Background

Medical students require training in order to perform clinical skills competently. Suboptimal performance of skills is associated with significant patient morbidity and mortality and increased healthcare costs [1,2]. For the purposes of this study, a skill is defined as “an ability to smoothly and adaptively carry out a complex activity acquired through deliberate, systematic, and sustained practice” (adapted from [3]). Clinical skills include technical skills such as clinical examination and invasive procedures, non-technical skills such as team – work and communication and cognitive skills such as clinical reasoning, decision-making and management (adapted from [4,5]). Michels et al. [5] found that the components of procedural steps, underlying knowledge and clinical reasoning are part of clinical skills learning and teaching and that underlying knowledge and clinical reasoning skills allow a clinical skill to be applied meaningfully.

For the purposes of this study, competence in a clinical skill is defined as the application of complex combinations of knowledge, performance, skills, values and attitudes at the level of being able to show how to perform the skill in a controlled setting rather than performing the skill in the workplace (adapted from [6,7]).

In this study, using Dreyfus and Dreyfus’ stages of skill acquisition [8], which range from novice to advanced beginner to competence to proficiency to expertise, we aimed to assist students in moving from the novice stage to the competent stage in skills acquisition for 3 clinical skills of history taking and physical examination. Dreyfus [9] notes that when people learn a skill, in order to reach competence, they need to learn to devise a plan or perspective that determines which of the elements of the skill must be treated as important and those that can be ignored. This aids understanding and decision- making. Dreyfus also points out that only at the level of competence is there for the first time an emotional investment in the choice of perspective leading to an action. Benner [10] found in nurses that emotional engagement in the form of experiencing deeply felt rewards or remorse as a result of their actions in the clinical setting seems to be necessary for the performer to learn from examples without rules.

Anderson proposed that 3 stages are involved in learning how to perform a skill [11]. These are the declarative stage, the knowledge compilation stage and the procedural stage. During the declarative stage general problem solving is used to interpret new information in a way that helps the learner deal with the skill required. With time, knowledge is compiled into higher order productions that apply the knowledge and increase efficiency in dealing with the learning task. This is followed by the procedural stage in which knowledge is incorporated into the procedures for performing the skill. Singley and Anderson proposed that exposure to the whole skill, for example via a demonstration, is most effective early on in learning a skill [12].

Despite models looking at stages of skills acquisition, there is little evidence as to the best way to teach clinical skills. Byrne et al. [13] examined publications on various methods of teaching the clinical skills of intubation, intravenous cannulation and central venous line insertion, but were unable to find any clear benefit for one instructional method over another.

Many instructional methods advocate demonstration of the clinical skill to be learned [14,15]. Demonstrations are a means of meeting the “being introduced to a topic or skill” and “getting to know it” steps of the learning model described by Ausubel [16]. The steps of this learning model are: being introduced to the topic or skill, getting to know it, trying it out, getting feedback and applying it. Hughes et al. [17] described how Ausubel’s learning model can be viewed as a cyclical rather than a linear process where the trying it out, getting feedback and applying it stages are a continuing cycle.

Lake and Hamdorf [15] advocate that demonstration should occur on a real patient to allow the learner to identify with a competent performance. Michels et al. [5] found in a survey that 100% of British doctors involved in teaching clinical skills agreed that “in learning a clinical skill it is important to have demonstration (modeling)”. Such demonstrations are commonly followed by students practicing the skill on real or surrogate patients, usually with supervision and feedback from tutors, meeting the “trying it out” and “getting feedback” stages of Ausubel’s learning model. Video demonstrations can help meet the early and also some of the later steps of the learning model described by Ausubel by providing a best practice exemplar for comparison in the “getting feedback” stage and also later in the revision stage. However, they clearly cannot replace immediate accurate feedback from a skilled observer.

The use of videos to demonstrate skills on real patients also fits well with Peyton’s 4 - step approach to teaching procedural and physical examination skills [14]. Peyton advocated silent demonstration first, followed by deconstruction, where the teacher demonstrates while explaining each step of the skill and then comprehension, where the teacher demonstrates while the learner describes the steps and then performance where the learner demonstrates while he or she describes the steps.

Viewed from an experiential learning perspective, videos can address the watching component of the “four step cyclical process” of experiential learning described by Kolb [18], the steps being “thinking, feeling, watching and doing”. Kolb notes that experiential learning can begin at any of these four steps.

Ericsson’s [19] concept of deliberate practice has been shown to be important in acquisition of skills. It has been found that individual differences, even amongst elite performers, are closely related to the amount of deliberate practice undertaken [19]. Improvements in performance of skills are associated with improved quantity and quality of practice.

As noted by Bradley and Postlethwaite [20], teaching of clinical skills, including demonstrations, can be time-consuming and resource intensive. It is uncertain as to whether students learn as effectively from video as from live demonstrations, although delivery of demonstrations of clinical skills through creation of enduring online video resources is cost-effective. Web - based learning has been shown to be cost effective in non-medical settings, with cost savings of up to 50% compared with traditional tutor led instruction [21].

Video resources also provide a reference for students during revision of skills. Kelly et al [22] found an unexpected revision benefit of videos demonstrating 3 procedural nursing skills, with high student uptake of the online videos during revision prior to the end of semester observed structured clinical examinations (OSCEs) and request by students for further online videos for skills to be learned in future years despite a very low rate of participation in their study to evaluate the impact of the videos on student learning.

Web – based video demonstrations allow for blended learning, a technique combining web-based or e-learning technology with traditional instructor led training [23]. Childs et al. [24] note that e-learning may be more effective when blended with traditional classroom based teaching.

Caveats regarding the use of videos as an adjunct for learning of clinical skills are that videos must be of high quality and demonstrate a competent performance and that those currently available via the internet are of variable quality [25]. Kingsley et al [26] showed that many early dental students lack the ability to critically appraise the quality of the information they access via the internet. Medical students are likely to be similar. They are potentially at risk of learning incorrect and potentially unsafe techniques through video, for example where breaches of aseptic technique are unwittingly demonstrated, unless they learn critical appraisal skills or alternatively, learning resources are first screened by faculty. Learners can easily be swayed by incorrect prompts. Beran et al [27] found that medical students given the assessment task of knee aspiration on a knee model were more likely to insert the aspiration needle at the incorrect site when they were provided with a knee model which had marks of aspiration at an incorrect site than when they were provided with an unmarked knee model (n = 31, 86.11% vs. n = 14, 58.33%, Fisher's exact test (1) = 5.93, p < 0.05, Cramer's ϕ = 0.31).

We conducted this study in view of the fact that although no clear benefit has been shown for one instructional method over another, we expected that exposure to high quality video demonstrations of clinical skills which students could refer back to was likely to improve student learning and hence competence at these skills. The aim of the study was to determine whether students randomized to access videos demonstrating endocrinology skills of history taking and physical examination perform these skills more competently than students randomized to standard revision resources. In this article the impact of access to endocrinology clinical video demonstrations on student performance is being reported.

## Methods

High quality instructional videos demonstrating performance of 3 common clinical skills in endocrinology were developed. These employed both real patients with clinical histories and signs and surrogate patients. The skills were:

1. Clinical history taking in diabetes mellitus (DMH)

2. Physical examination for lower limb complications in diabetes mellitus (LLE)

3. Physical examination for signs of thyroid disease (TE)

The videos were designed to meet the 2^nd^ year medical student endocrinology learning objectives for the Sydney Medical Program. They could be used either as an introduction to the topic or for revision. For the clinical examinations, the videos demonstrated the examination process and technique in a surrogate patient without clinical signs, showing a normal examination, and then illustrated specific clinical signs in patients with abnormalities. The DMH video demonstrated history taking in 3 patients, illustrating features of both type 1 and type 2 diabetes mellitus. The skills were performed on each video by a single endocrinologist (EH) and for each video, the technique, rationale and important findings were explained and demonstrated. Subtitles were added in the editing process to highlight important points and provide additional information.

All patient participants were provided with an information sheet and gave written informed consent.

Second year medical students from 3 clinical schools in the University of Sydney Medical Program were recruited via email invitation. They were invited because they undergo their first exposure to clinical endocrinology during their second year over a period of 4-6 weeks through weekly small group bedside tutorials focused on clinical histories and examinations of patients with endocrine conditions. They usually have 2 tutorials per week, one addressing history taking and one addressing physical examination in endocrinology. The tutorials provide opportunities for students to learn and practice endocrinology skills with real and surrogate patients, while being observed and receiving feedback from a tutor. These tutorials are preceded by a single live lecture demonstration by a faculty member (EH) to the whole year of students, which includes a demonstration on a surrogate patient of two of the skills addressed by the newly developed videos: examination for lower limb complications in diabetes and examination for signs of thyroid disease. Important elements for the third skill demonstrated in the videos, history taking in diabetes, are discussed in the full year session but not demonstrated with a patient. This usual clinical learning in endocrinology, both the live demonstration and discussion delivered once to the entire second year and the bedside tutorials, covers the key features of the criteria for each assessment task in the observed structured clinical examination assessments detailed below.

Students gave written informed consent to participate in the study. They signed a confidentiality agreement at recruitment precluding them from sharing access to or information from the videos with anyone other than students enrolled in the study who were attending the same clinical school as them on the same day. This was in order to allow them to practice the skills with their peers. The study was approved by the Human Research Ethics Committees of the Sydney South West Area Health - Concord, the Northern Area Health Service, the Central Sydney Area Health Service and the University of Sydney.

Student participants were randomized to gain access (training video or TV group), or not (revision as usual or RAU group), to each of the instructional clinical videos within a few weeks following completion of their clinical tutorials in endocrinology. Student participants were advised to revise the clinical skills using any resources available to them, including any online video resources they could access. They were informed that they would be assessed on all 3 skills in an OSCE format two weeks after randomization. Each assessment task was in the form of a 7-minute OSCE in a similar format to clinical assessments they had already been exposed to in the first year of the medical course. Students who were not participating in the study did not undergo the OSCE assessments for this study. However, all 3 skills are clinical skills that could potentially be examined in the end of year barrier OSCE assessment, as they are part of the core curriculum.

Randomization occurred according to day of student attendance at their clinical school, which can be on one of two weekdays. Students accessed the videos online via their usual individual student log on identification for the university website. They could download videos to which they had been randomized to computer, iPhone or iPod.

In the OSCEs, students were assessed on taking a history of diabetes mellitus from an actor who had learned a script and examination on surrogate patients without clinical signs for both of the physical examination tasks. At the start of each assessment station, each student was given brief written instructions specifying the skill to be performed, without prior knowledge as to whether clinical signs were present. For each assessment task students were observed by either one assessor or by two assessors marking the same performance independently. Assessors were blinded to student randomization status. Students were rated on both a criterion - referenced checklist for each task, with descriptors for each criterion of “attempted and correct”, “not attempted” or “attempted but not correct” and on a global score of “satisfactory”, “not satisfactory” or “borderline”, based on the judgment of the assessor. They received a mark for a criterion only where the criterion was rated as “attempted and correct”. The criteria for each assessment task were determined by consensus of a panel of endocrinologists prior to production of the videos. Each video demonstrated competent completion of all the criteria determined by the panel of endocrinologists for that clinical skill. However the criteria were not necessarily addressed in exactly the same order as in the OSCE marking sheet. The same criteria had also been addressed in the live demonstration of TE and LLE to the whole student year at the beginning of the endocrinology block of learning. The TE and DMH assessments were developed by adapting pre-existing university clinical assessments. The thyroid station had 25 criteria, the diabetes history station 22 criteria and the lower limb complications station 18 criteria.

Immediately following completion of all 3 assessment tasks, students were asked to complete a brief survey questionnaire for each of the 3 clinical skills to explore their perceptions on the utility of the learning resources they accessed. Data on the number of times each video resource was accessed were retrieved through the website.

Following completion of the study, all students in the 2^nd^ year of the medical program were given access to all three videos.

### Statistical analysis

Assessment data were analyzed using chi-square tests with a continuity correction by a statistician who was blinded as to intervention group identity. Data were analyzed using SPSS version 17.0. Where there were 2 examiners at a station, only one examiner’s rating was analyzed. The endocrinologist’s ratings were chosen in preference to the non-endocrinologist’s. Where there were 2 endocrinologists at a station, the examiner who had examined more students was selected for analysis. Where there were 2 examiners at a station, the degree of agreement between the examiners was analyzed.

## Results

Twenty-three students participated in the study. This represented 12.2% of the 2^nd^ year student cohort from the 3 clinical schools involved in the study. Twenty two students (11.6% of the 3 clinical school year cohort) were assessed, as one student was unable to attend the assessment. Twelve students (54.5% of participants) were randomized to both of the diabetes videos, and ten students (45.5%) to the TE video.

The assessment team comprised 4 endocrinologists, one advanced trainee in endocrinology, one oncologist and one geriatrician. Non - endocrinologists were teamed with endocrinologists to mark independently at stations. The videos ranged in duration from 33-48 minutes.

### Performance in assessment of the clinical skills

Students randomized to view the diabetes videos performed significantly better in the diabetes assessments than students who were not randomized to view the videos. For the LLE assessment task, 91.7% (n = 11/12) of students who had viewed the video were judged globally as having performed the task satisfactorily ie competently compared with 40% (n = 4/10) of those who had not viewed the video (p = 0.024). The difference in performance between the 2 groups was 51.7% (95% confidence intervals: 17.6 - 85.8%). For the DMH assessment task, 83.3% (n = 10/12) of students who had viewed the video were judged globally as having performed the task satisfactorily in the assessment versus 20.0% (n = 2/10) of those who had not viewed the video (p = 0.007). The difference in performance between the 2 groups was 63.3% (95% confidence intervals 30.7-95.9%). For the DMH video, in order to capture the maximum number of students, the results from 3 different examiners were included for the analysis, as most students had only one examiner at this station, rather than 2 examiners marking independently. Results were similar in degree and significance when re-examined using only the data from the endocrinologist who had examined most students at this station.

For the TE assessment task performance standard was uniformly high. There was no difference in the standard of performance of students randomized to view the TE video compared with those not randomized to view it, either on examiner global judgment or on any of the 25 criteria. Ninety percent (n = 9/10) of students who had viewed the video were judged globally as having performed satisfactorily compared with 100% (n = 12/12) of those who had not viewed the video (p = 0.926).

In the LLE assessment, students who had viewed the video performed significantly better on 4 out of the 18 individual criteria (Table 1). These criteria were: palpating the dorsalis pedis pulse at the correct site bilaterally, checking for light touch sensation using correct technique, performing knee reflexes correctly and indicating a plan to check lower limb motor function.

**Table 1:** Lower limb examination assessment criteria showing significant performance differences

**Description of criterion**	**Video group % correct (n) (Total n = 12)**	**Control group % correct (n) (Total n = 10)**	**Difference in %**	**P value**	**95% CI lower**	**95% CI upper**	**% Examiner agreement**
Global judgment	91.7 (11)	40.0 (4)	51.7	0.024	17.6	85.8	72.7
Palpates dorsalis pedis pulse correctly bilaterally	91.7 (11)	30.0 (3)	61.7	0.009	29.3	94.1	76.2
Checks light touch sensation correctly	100.0 (12)	30.0 (3)	70.0	0.002	41.6	98.4	57.2
Performs knee reflexes	83.3 (10)	30.0 (3)	53.3	0.01	17.9	88.7	95.2
Indicates a plan to test motor function	72.7 (8)	0 (0)	72.7	0.003	46.4	99.0	95.0

Students randomized to view the DMH video performed significantly better in 3 of the 22 criteria (Table 2) than those who had not been randomized to view the video. These criteria were: asking about exercise, history of ischaemic heart disease and history of cerebrovascular disease. Of the students who reviewed the DMH video, all (12/12) enquired whether the patient had retinopathy and when their last formal eye assessment was performed, compared with 60% (6/10) of students who had not viewed the video (p-value = 0.05).

**Table 2:** Diabetes history assessment criteria showing significant performance differences

**Description of criterion**	**Video % students correct (n) (total n =12)**	**Control % students correct (n) (total n = 10)**	**Difference in %**	**P value**	**95% CI lower**	**95% CI upper**	**Examiner agreement %**
Retinopathy and time of last eye check	100.0 (12)	60.0 (6)	40.0	0.053	9.6	70.4	76.9
Exercise	75.0 (9)	20.0 (2)	55.0	0.036	20.1	89.9	50
Ischaemic heart disease	75.0 (9)	40.0 (4)	35.0	0.004	-4.0	74.0	75
Cerebrovascular disease	75.0 (9)	20.0 (2)	55.0	0.03	20.1	89.9	100
Global judgment	83.3 (10)	20.0 (2)	63.3	0.007	30.7	95.9	87.5

### Examiner agreement on marking

Two examiners examined all but one student concurrently and independently for the LLE and TE assessment tasks. For the DMH assessment task only 8 (36.4%) of the students had two examiners. Examiner agreement was high for all of the stations, especially for the global judgment of student performance. For the lower limb examination, examiner agreement on marking of students was ≥ 80% for 77.8% (ie 14/18) of the criteria. For the global judgment of student performance, agreement was 80.2%. For the thyroid station examiner agreement was ≥ 80% for 92% (ie 23/25) of the criteria with agreement on global judgment of performance 95.3%. For the diabetes history taking, examiner agreement was lower at 68% with agreement on 15/22 criteria for the 8 students examined by 2 examiners. However, agreement on the global judgment of student performance was high at 87.5%.

### Student evaluation of the video resources

Twenty one students (95.5%) completed the evaluation questionnaires for each station. All students randomized to the diabetes videos reported accessing them. One student randomized to the TE video reported not accessing it. The LLE and DMH videos were viewed up to 3 times on student self-report and the TE video once or twice (Table 3).

**Table 3:** Number of times the video resources were viewed by students

**Times viewed**	**Lower limb**	**Diabetes Hx**	**Thyroid**
1	25.0% (3/12)	25.0% (3/12)	66.7% (6/9)
2	33.3% (4/12)	50.0% (6/12)	33.3% (3/9)
3	41.7% (5/12)	25.0% (3/12)	

### Student perception of the value of the endocrine videos in their learning

Eleven of 12 students (91.7%) who viewed both the LLE and DMH videos strongly agreed on a 5 point Likert scale (strongly agreed – tend to agree - neither agree or disagree - tend to disagree - strongly disagree) that the videos were useful for them in learning the clinical skill. For the thyroid examination video, 55.6% of students (5/9) who completed the evaluation and had viewed the TE video strongly agreed that it was useful for them in learning the clinical skill and 44.4% (4/9) tended to agree that it was useful.

### Student time spent practicing the clinical skills

The median amount of time students reported practicing the clinical skills with real or surrogate patients was 33 - 44 minutes for each skill but there was a broad range from 0 -14 minutes to greater than 3 hours.

### Frequency and mode of access of the endocrine videos

There were a total of 87 hits on the endocrine video website. Forty seven were by iPhone or iPod download and 40 by computer mp4 file download. Students accessed the TE video more frequently as an iPhone download (18 hits) than as an mp4 file to be viewed on computer (2 hits). There were a similar number of hits via each method for the diabetes videos: for DMH 15 by iPhone or iPod and 21 by computer mp4 file; for LLE 14 by iPhone or iPod and 17 by computer mp4 file.

### Student perception of most useful aspects of the videos

Students reported that the most useful aspects of the videos were being able to observe the correct technique, the use of real patients, seeing how a professional approaches the patient and performs the skills, the ability to observe normal and abnormal clinical signs, having multiple patients and the ability to download the videos onto an iPhone.

## Discussion

This study found that students randomized to view the clinical diabetes videos performed significantly more competently than students who had not viewed the videos. There was no difference in performance of students on thyroid examination whether randomized to view the TE video or not. It is surprising that there was such a major difference in assessed competence for the diabetes assessment tasks as, although the videos were an additional resource, the material they covered was not essentially different in content from the material students had been exposed to previously. The ostensible differences between the videos and the live demonstrations were that the videos showed a close up view of technique with explanation of both technique and the significance of findings on history and physical examination, involved a number of real patients with physical signs, which gave repetition and possibly meaning and that students were able to view them at their own speed and also to review them at leisure.

The differences in competence of performance of diabetes - related assessment tasks are clinically significant differences, which may translate to meaningful differences in clinical performance in the workplace and potentially improved patient outcomes. Diabetes mellitus is common with a prevalence of 7.5% in Australia in 2000 [28] and its complications are a significant cause of morbidity and mortality [29]. Insight into diabetes management and detection of complications in an individual patient through careful history taking and physical examination can inform appropriate management which can significantly improve patient outcomes.

However, the students underwent a competency- based assessment which measures what they do in a controlled representation of practice, such as an OSCE, rather than a performance -based assessment, measuring what they do in actual professional practice [30] so their performance in assessment of patients with diabetes in the workplace may not be superior to that of the control group. However, an OSCE type of assessment was selected for this study as it is practical to deliver and allows for standardization of conditions for students, facilitating comparisons between students. Workplace assessment is less easily standardized for large numbers of students and for assessors and is less practical in terms of the difficulty of scheduling times for assessment, although it is probably a more valid assessment of clinical skills.

It is unclear why there was no difference in performance between students randomized to the thyroid video and those who were not. Both groups demonstrated a high level of competence. The study would not have had the power to detect small differences in performance between groups. However, the lack of difference between groups may also relate to the fact that there were already a number of thyroid videos online [31-33], some of which were of fairly high quality. In addition, students may have already practiced the thyroid examination more than the other skills and received tutor feedback on their performance, as it was one of the formative assessment exercises they could complete during their endocrinology clinical bedside tutorials.

In contrast, there are some factors that may explain the greater competence of the TV student group at the diabetes related clinical skills. Only one video could be found online demonstrating physical examination for lower limb complications in diabetes at the time of the study [34], although some have been added since. This video was not comprehensive and seemed aimed more for a patient than for a medical student or medical practitioner audience. There were no formative assessment tasks relating to physical examination for lower limb complications of diabetes or for taking a full history of diabetes in the students’ curriculum. The complexity of the diabetes related skills may have been higher than those required for thyroid examination, requiring more advanced psychomotor skills which may potentially have made the videos more useful. Silverman et al. [35] argue that communication involves a different type of content than other practical clinical skills or cognitive learning. It may be that video demonstration assists with this type of communication skills learning. In addition, the practical significance of particular findings on history and examination, for example asymptomatic hypoglycaemia, was stressed in the diabetes videos, whereas this may have been less so for the thyroid video, giving the students a means to make the diabetes information memorable. It could also be that there is more variation in the way that clinicians teach lower limb examination in diabetes, although the recommended student textbook for clinical examination [36] explains physical examination in diabetes much as demonstrated in the video with the exception of the use of a 10 gram monofilament, which is not described. However, the textbook description is far less detailed than the video. Despite this, there was no significant difference in competence of students between groups in use of the 10 gram monofilament.

The majority of the literature looking at use of video in teaching clinical skills has used videos demonstrating procedural skills rather than history and examination skills. However Maloney et al. [37] looked at physiotherapy student learning of two clinical skills: initial assessment and subsequent patient education for an acute cervical spine complaint and initial assessment and treatment of an inpatient with a vestibular problem. Students were randomized to learn the skill via three methods. Group 1 had traditional face- to - face tutorials with a live demonstration of the skill by class tutors followed by an opportunity to ask questions of the tutor and then a 30 minute supervised practice session of the skill. Group 2 were given a pre-recorded video tutorial delivered in a group setting, demonstrating the skill with a patient, then repeating the skill with commentary explaining the tutor’s clinical reasoning. The video instructed students to conduct 10 minutes of practice with a student colleague. Group 3 were given a written online simulated patient scenario requiring the student to utilize the practical skill of interest. Group 3 students had to source the knowledge to complete the skill and make and submit a brief video of themselves demonstrating the skill to receive brief written tutor feedback. All 3 groups performed the skills equally well in an OSCE setting with assessors blinded to student allocation. However, the students were significantly more likely to agree or strongly agree that the video based methods helped them learn the skill compared with the traditional teaching method (p = 0.002).

Holland et al. [38] showed that nurses given unlimited access to an online best practice exemplar video of medication administration in addition to standard lectures and skills classes usually given to teach this skill performed significantly better on an observed structured clinical assessment on medication administration (p = 0.021). They were also more satisfied with the teaching than those who attended only the standard learning activities.

Marteau et al. [39] trained students in communication skills through six small group seminars over a year. The sessions involved basic communication skills and addressed areas of difficult communication. The techniques used included showing videos, role-play with and without video feedback, and feedback on students’ videotapes of their interviews with patients. At the end students were assessed through a simulated patient encounter where they took a history of the presenting complaint. Participating students were compared with students who had not received communications skills training. Communication skills training did improved competence to some extent but student gender was a better predictor, with female students rated as more competent, empathic and warmer than male students.

The literature suggests that video educational resources may be effective in delivering educational content in a more time efficient way than traditional textbook resources. Steedman [40] found that students randomized to learn about acute eye conditions via textbook excerpt reading versus viewing a video performed equally well on multiple choice assessment of knowledge despite less time, a mean of 8 minutes, spent learning from the video compared with 29 minutes mean for textbook reading (p = 0.0003).

Video resources used in revision of a clinical skill may assist in maintaining competence at performing the skill over time. This has been shown for medical students performing female and male catheterization 3 months after learning the procedure [41] and for subcuticular suturing one week after learning the technique [42].

Video can also, by the fact that it can be viewed repeatedly without any further input of time from teaching staff, give students the opportunity to become familiar with an area in their own time and at their own pace. Levitan et al. [43] showed that paramedics asked to watch a 26 minute videotape of laryngoscopy thrice in addition to an existing airway training program had higher mean rates of success for first intubation attempt at 88.1% compared with 46.7% for historical controls.

Students seem to prefer learning by watching videos than by standard instructional methods [38]. Ninety six percent of University of Dundee medical students [44] rated e-learning resources introduced to their cardiology program as probably or definitely of value. Almost all of the students found the animations, the self-assessment exercises and the video demonstrations valuable. They perceived the advantages of the resources as being “the ease of access and choice of time, pace and place for learning”. Veterinary students at the University of Nottingham [45] cited the strengths of online video resources as being: teaching enhancement, accessibility, technical quality and video content.

Ruiz et al. [46] note that web-based or e-learning is at least as effective in the medical setting as traditional instructor related methods such as lectures. McKimm [47] observes that web -based programs may encourage independent and active learning and can be an efficient way to deliver course materials. However, e-learning can also be problematic. Technology is the main barrier to the use of effective web based learning materials, for example poor access and slow downloading, rather than the design of the learning material [47]. Chumley-Jones et al. [48] found that the main predictor of satisfaction with web-based learning in medicine was download speed. High download speeds were associated with high satisfaction and the converse was also true. The number of hits on the online videos in this study may have been a marker of difficulty in downloading the videos or alternatively, of students downloading videos to multiple devices.

A lack of interactivity with the web-based video or other material can also be a problem with e-learning [24]. However, the way in which the videos were used in this study was as an additional resource to complement the students’ face-to-face clinical skills practice, in a blended learning format. This kind of blended approach, with a web-based and a face-to-face component seems the most preferred by students [49]. McKimm [47] notes that web based video demonstrations of clinical skills can be particularly useful to support clinical teaching when learners are geographically dispersed.

The strengths of this study are that competence was assessed in an observed clinical assessment of the skill, testing at the highest level that can practically be performed without assessment in the workplace [50]. Another strength was that assessors were blinded to student randomization status and marked independently, yet had a high degree of agreement on both individual criteria and more particularly on global judgment scores.

Limitations of the study are its small size, which gives wide confidence intervals for the difference in performance between student groups and raises the question of its generalizability, as participating students may not be representative of the whole student cohort. Randomization of students by day of attendance at their clinical school is a potential weakness, as it may have led to grouping of more or less competent students on particular days. Studies involving larger numbers of medical students are needed to confirm the results.

## Conclusions

Videos demonstrating clinical skills can be a valuable adjunct in revision and can improve learners’ competence in the clinical skills demonstrated, over and above competence seen in learners provided with face-to-face demonstration and supervised and unsupervised practice alone. Online video demonstrations provide an enduring resource for students and medical practitioners to refer back to and allow flexible on-demand learning. They can be made readily accessible through downloads to smart phones and tablets. This facilitates their use in a wide variety of clinical settings as a resource for the education of health practitioners. They are easily scalable, so once developed, can be provided to any number of users in a cost-effective manner.

## Abbreviations

TV: On-line skills training videos; RAU: Revision as usual; DMH: Clinical history taking in diabetes mellitus; LLE: Physical examination for lower limb complications in diabetes mellitus; TE: Physical examination for signs of thyroid disease; OSCE: Observed structured clinical examination.

## Competing interests

The authors declare that they have no competing interests.

## Authors’ contributions

EJH was chiefly responsible for the conception, design and coordination of the study, development of the videos and drafting of the manuscript. TL was involved in the developing, filming and editing the videos, analyzing the data and revising the manuscript. JNC, DLL and ST were involved in development of the OSCE assessments, acted as assessors in the OSCEs and were involved in revising the manuscript. SC was involved in assessment in the OSCEs and revising the manuscript. All authors read and approved the final manuscript.

## Authors’ information

Emily Hibbert is an Associate Professor in Medicine at the Sydney Medical School - Nepean. She is an endocrinologist with a strong interest and involvement in medical education and medical education research. Tim Lambert is a Professor of Psychiatry at the Sydney Medical School – Concord. He has a long-standing involvement in medical education and research interests in psychosis and medical education. John Carter is a Clinical Professor of Endocrinology at the Sydney Medical School – Concord with a major interest in student teaching and general endocrinology. Diana Learoyd is an Associate Professor in Medicine at the Sydney Medical School – Northern. She is an endocrinologist with a research interest in the thyroid and is involved in medical education. Stephen Twigg is a Professor of Medicine at the Sydney Medical School- Central. He is an endocrinologist with research interests in diabetes and is involved in medical education. Stephen Clarke is a Professor of Medicine at the Sydney Medical School- Northern. He is an oncologist with research interest in colorectal cancer and involvement in medical education.

## Pre-publication history

The pre-publication history for this paper can be accessed here:

http://www.biomedcentral.com/1472-6920/13/135/prepub
